# A FRET Approach to Detect Paraoxon among Organophosphate Pesticides Using a Fluorescent Biosensor

**DOI:** 10.3390/s22020561

**Published:** 2022-01-12

**Authors:** Andreia C. M. Rodrigues, Maria Vittoria Barbieri, Marco Chino, Giuseppe Manco, Ferdinando Febbraio

**Affiliations:** 1 Institute of Biochemistry and Cell Biology, CNR, Via P. Castellino 111, 80131 Naples, Italy; mariavittoria.barbieri@ibbc.cnr.it (M.V.B.); giuseppe.manco@cnr.it (G.M.); 2 Department of Chemical Sciences, University of Naples “Federico II”, 80126 Naples, Italy; marco.chino@unina.it

**Keywords:** biosensor, thermostable enzyme, organophosphates, *Alicyclobacillus acidocaldarius* EST2, fluorescence resonance energy transfer (FRET)

## Abstract

The development of faster, sensitive and real-time methods for detecting organophosphate (OP) pesticides is of utmost priority in the in situ monitoring of these widespread compounds. Research on enzyme-based biosensors is increasing, and a promising candidate as a bioreceptor is the thermostable enzyme esterase-2 from *Alicyclobacillus acidocaldarius* (EST2), with a lipase-like Ser–His–Asp catalytic triad with a high affinity for OPs. This study aimed to evaluate the applicability of Förster resonance energy transfer (FRET) as a sensitive and reliable method to quantify OPs at environmentally relevant concentrations. For this purpose, the previously developed IAEDANS-labelled EST2-S35C mutant was used, in which tryptophan and IAEDANS fluorophores are the donor and the acceptor, respectively. Fluorometric measurements showed linearity with increased EST2-S35C concentrations. No significant interference was observed in the FRET measurements due to changes in the pH of the medium or the addition of other organic components (glucose, ascorbic acid or yeast extract). Fluorescence quenching due to the presence of paraoxon was observed at concentrations as low as 2 nM, which are considered harmful for the ecosystem. These results pave the way for further experiments encompassing more complex matrices.

## 1. Introduction

Organophosphates (OPs) are a class of neurotoxic compounds including molecules with diverse applications from insecticides to herbicides or nerve agents [1]. OPs’ high efficiency against target plagues and their low persistence has led to their wide use in urban and agricultural areas, and they are commonly found in environmental samples [2,3,4,5]. OPs act by inhibiting the activity of acetylcholinesterase (AChE) enzymes, leading to muscular dysfunction in target organisms [6]. Nevertheless, due to similarities in the AChE family among diverse groups of organisms, OPs also pose a risk to non-target animals, including humans [7,8]. The neurotoxic action in children is of special concern to the whole of society, as children are generally more vulnerable because their brains are still developing. The adverse effects may also be intensified because the ratio of pesticide dose to body weight is higher than in adults, and children have lower detoxification rates [9,10]. The widespread presence of OPs in the environment has led to a common effort to monitor their concentrations in environmental (water, soil, air and food) and human (urine, blood and tissue) samples.

The detection of OPs in different environmental samples is usually achieved via powerful analytical techniques such as gas and liquid chromatography combined with mass spectrometry measurements (GC- and LC-MS) [11,12,13].

However, there are several alternative approaches that have been developed in the past decades to support the current technologies, in order to increase the use of, and speed up, OP monitoring. In particular, the development of sensors and biosensors for the detection and quantification of OPs in different environments (water, soil, food, etc.) and human samples (blood, urine, tissue, etc.) is growing [14].

Among these, enzymatic biosensors could be promising as one of the methods proposed as a faster, cheaper and simpler technique [15]. Some attractive features of these biosensors can be summarised in a few points: (i) less sample required, (ii) fast response time, (iii) high sensitivity, (iv) high specificity, (v) the possibility of being used in the field and (vi) the possibility of real-time measurement of samples. In particular, fluorescence-based sensors provide high sensitivity, robust signal-to-noise ratios and rapid response times, even in real-world environments (see review in [16]).

However, some limitations have also been observed, such as the specificity and the thermal dependence of the enzymatic activities proposed as bioreceptors [14]. Hence, there is a constant need for new, more efficient and stable enzymatic activities for use as bioreceptors. Esterase 2 from *Alicyclobacillus acidocaldarius* (EST2) is a carboxylesterase belonging to the hormone-sensitive lipase family, which has recently been explored for applications as bioreceptors for the detection of OPs due to its thermostability and specificity with respect to these compounds [17,18].

Using a fluorogenic substrate, we were able to measure with high efficiency the residual activity of EST2 after covalent binding to the OPs [19,20], improving the measurements carried out with colorimetric substrates [18]. However, this approach remains an indirect determination of the binding of OPs to the protein. Thus, it presents a limitation due to the need to add the EST2 substrate into the analysis, making it difficult to apply for real-time measurements. This limitation can be overcome by exploiting the quenching of the intrinsic protein fluorescence, which is shown by the protein tryptophan residues due to their electronic delocalisation of the indole aromatic ring. The intrinsic fluorescence of EST2 tryptophan was previously explored to detect the binding of OPs using fluorescence-based methods [21]. However, fluorescence quenching is highly dependent on substrate concentration. Therefore, a mutant of EST2 with the serine 35 replaced by a cysteine residue (EST2-S35C) was developed to bind an extrinsic fluorescent probe. The addition of a fluorescent probe has several advantages. It increases the sensitivity of the bioreceptor to slight environmental changes, allowing for the detection of conformational variations of macromolecules or their interactions and allowing binding with other analytes through the measurement of the displacement of the dyes [22].

Fluorescence-based biosensors have been proved to have a high degree of sensitivity and specificity, allowing for real-time detection of target molecules in solution at environmentally relevant levels. We previously proposed their use in a flow cell of labelled EST2-S35C for real-time detection of OPs [23]. Nevertheless, these biosensors may be limited due to the high concentration of fluorophore required, leading to quenching due to aggregation [24], or by the complexity of the environmental sample with other organic compounds emitting fluorescence at similar wavelengths. Förster resonance energy transfer (FRET)-based sensors may be an effective alternative to overcome these problems.

That said, the main objective of this study was to evaluate the use of labelled EST2-S35C as an enzymatic bioreceptor, coupled with FRET measurements for the detection of OPs in complex mixtures. To achieve this, we assessed: (1) the stability of the bioreceptor under different pH conditions; (2) the efficacy of FRET measurements in avoiding interference from other organic compounds in the medium; (3) the selectivity of EST2-S35C towards paraoxon, compared with thio-OPs.

Briefly, good stability and replicability of FRET measurements applied to the studied bioreceptor were observed. Furthermore, the specificity of EST2-S35C towards paraoxon was also clearly observed in the presence of thio-OPs. Therefore, applying FRET measurements using EST2-S35C as a bioreceptor is promising regarding the complexity of biological matrices.

## 2. Materials and Methods

### 2.1. Reagents

Diethyl-p-nitrophenyl phosphate (paraoxon), diethyl p-nitrophenyl thiophosphate (parathion), isopropylmethylpyrimidyl diethyl thiophosphate (diazinon), tributylphosphine (TBP), tris(hydroxymethyl)aminomethane hydrochloride (Tris/HCl), dimethyl sulfoxide (DMSO), glucose, ascorbic acid and selected yeast extracts were purchased from Sigma-Aldrich (Merck & Co., Inc., Kenilworth, NJ, USA). Bio-Rad dye reagent was purchased from Bio-Rad Laboratories, Inc., Hercules, CA, USA. 5-((((2-iodoacetyl)amino)ethyl)amino) naphthalene-1-sulfonic Acid (1,5-IAEDANS) was purchased from Molecular Probes (Thermo Fisher Scientific, New York, NY, USA). All the reagents were of analytical grade.

### 2.2. Over-Expression and Purification of EST2-S35C

The mesophilic host *E. coli* strain BL21 (DE3), already available in our lab [25], was selected to over-express EST2-S35C, and was extracted and purified as previously detailed by Carullo et al. [23], with slight modifications. As previously described, TBP was added as a reducing agent to preserve the mutated cysteine in the reduced form. In addition, minor modifications to the extraction and purification steps were implemented. In particular, the protein was extracted using a sonication step performed on a Branson Sonifier Sound Enclosure Model SSE-1 (3 cycles of 40 s ON/30 s OFF, pulses at 50% power output intensity), in a water/ice bath at 4 °C. This step was followed by ultracentrifugation (80,000 g at 4 °C for 30 min) to remove the cell debris. After the thermo-precipitation/ultracentrifugation steps, gel filtration was performed using a Sephadex G-25 column (GE Healthcare Bio-Sciences AB, Uppsala, Sweden), achieving >95% purity of the enzyme. The final protein concentration was calculated after the Bradford method with bovine ɣ-globulin as the standard [26].

### 2.3. EST2-S35C Labelling and Inhibition Efficiency

The polar solvent DMSO was used to dissolve IAEDANS at a final concentration of 20 mM. As described in Carullo et al. [23], EST2-S35C (15 × 10^−9^ moles), in 25 mM Tris/HCl buffer at pH 7.5 and 1 mM TBP, was conjugated with IAEDANS (from 10- to 200-fold molar excess), and incubated overnight in the dark at room temperature. The excess of the probe was removed by dialysis against a 25 mM Tris/HCl buffer at pH 7.5 at room temperature in the dark, using a QuixSep Micro Dialyzer (Creative Biomart, New York, NY, USA). The protein probe concentration was determined using the Bio-Rad dye reagent, as described by Bradford et al. [26].

The inhibition efficiency of paraoxon on EST2-S35C was determined as described in Carullo et al. [23]. Briefly, aliquots of 2.1 pmol of EST2-S35C were incubated at room temperature in 25 mM Tris/HCl at pH 7.4 in the presence of increasing concentrations of paraoxon in the range from 0 to 2 pmol. The residual enzymatic activity was measured by assaying sample aliquots on 0.2 mM p-nitrophenyl-hexanoate in 40 mM sodium phosphate buffer at pH 7.0, monitoring the increase in absorbance at a wavelength of 405 nm by the release of p-nitrophenol as a reaction product.

### 2.4. Förster Resonance Energy Transfer Measurements

In brief, the Förster theory presents FRET as the transference of an exciton from a donor to an acceptor at a proximity of 0–10 nm, as a molecular non-radiative process. For this process to occur, a superposition is necessary between the donor absorbance spectrum and the acceptor emission spectrum [27].

Aliquots of a stock solution of 1.0 µg/µL (~30 pmol) EST2-S35C, labelled with IAEDANS, were used for FRET measurements at different labelled-protein amounts (0, 3, 9, 12, 15, 30, 90, 120 and 150 pmol) in a 450 µL final volume of 25 mM Tris/HCl (pH 7.5) buffer.

Fluorescence spectroscopy measurements were performed on a Jasco FP-8200 (JASCO, Tokyo, Japan) spectrofluorometer. All experiments were performed in triplicate. The emission spectra of the fluorescent probe conjugated with EST2-S35C were recorded in a 1 cm optical path length cuvette, in the range from 300 to 550 nm using an excitation wavelength of 290 nm, 1 nm step resolution and 500 nm/min scan speed, with 3 accumulations. Data were acquired using Spectra Manager 2.09 software (JASCO, Tokyo, Japan).

### 2.5. Interference in FRET by pH and Chemicals

The stability of the EST2-S35C FRET signal intensity was evaluated under different pH values. For this purpose, 25 mM NaH_2_PO_4_ buffer at pH 7.0 and 25 mM Tris/HCl buffer at pH 7.5 and 8.5 were used to individually incubate 67 nM (30 pmol/450 µL) EST2-S35C for 1 min at room temperature. In addition, to evaluate the possible interference with respect to the FRET intensity of organic compounds present in complex samples, glucose (in the range from 1 to 6 mM), ascorbic acid (in the range from 1 to 6 mM) and yeast extracts (in the range from 1 to 3 mg/mL) were individually tested with EST2-S35C (67 nM) in the FRET measurements.

### 2.6. Detection of Paraoxon by FRET

Measurements of the FRET quenching of inhibited EST2-S35C (67 nM) were performed by adding increasing concentrations of OPs to the solution. In particular, stock solutions (10 mM) of paraoxon, parathion and diazinon in DMSO were used. To set up the quenching measurements, increasing concentrations of paraoxon (0, 2.2, 11.1, 22.2, 33.3, 44.4, 55.6, 66.7 and 77.8 nM), were tested in the solution (450 µL) containing 67 nM EST2-S35C, by incubating for 1 min before acquisition of the emission spectra. FRET measurements on single or complex solutions of OPs (450 µL) were performed by adding to EST2-S35C (67 nM) increasing concentrations (in the range from 0 to 26.7 nM) of paraoxon, parathion and diazinon, individually, in a 1:1 mixture of parathion/diazinon and in a 1:1:1 mixture of paraoxon/parathion/diazinon.

### 2.7. Data Analysis

The linearity of the data was assessed, and the robustness of replicates was tested using the F test with *p* < 0.05. The limit of detection (LOD) and limit of quantification (LOQ) were calculated using the equations LOD = 3 × (SD/slope) and LOQ = 10 × (SD/slope), respectively. One-way ANOVA was used to test for the pH effect on bioreceptor stability. The relative standard deviation (RSD) was calculated for each data set and presented in Table S2. Statistical analysis was performed using the GraphPad Prism version 7.0 software for Microsoft Windows (GraphPad Software, La Jolla, CA, USA).

### 2.8. In Silico Preparation of Mutated and Labelled EST2-S35C 3D Structure

The 3D crystallographic structure of EST2, resolved at 2.6 Å (PDB-ID 1EVQ), was retrieved from the RCSB PDB online database (https://www.rcsb.org/ (accessed on 7 December 2021)) [28] and repaired using the PyMOL software [29], by: (i) replacing the seleno-methionine derivative, (ii) removing the 4-(2-hydroxyethyl)-1-piperazine ethanesulfonic acid molecule bound to the catalytic serine 155 and (iii) removing the other heteroatoms, such as water molecules. The web-based platform CHARMM-GUI (http://www.charmm-gui.org (accessed on 7 December 2021)) was used to prepare the S35C mutant of the EST2 3D structure, replacing the serine residue in position 35 with a cysteine residue. The generated structure was optimised using the CHARMM software [30], performing a basic energy minimisation through an initial 25–50 steps of the steepest descent (SD) algorithm (to remove bad van der Waals contacts) and subsequently 1,000,000 steps of the Newton–Raphson method (ABNR) to remove potential problems such as collisions and non-physical contacts/interactions. Using the Avogadro software (https://avogadro.cc (accessed on 7 December 2021)), the 3D structure of the fluorescent IAEDANS probe was manually added to the sulfur group of the cysteine 35.

### 2.9. Docking Analysis

The molecular docking analyses were carried out on the optimised 3D structure of EST2-S35C bonded to IAEDANS, using AutoDock Vina software [31], employing the Broyden-Fletcher-Goldfarb-Shanno algorithm, significantly improving the average accuracy of the binding mode predictions compared to AutoDock 4. In order to determine the grid size and add polar hydrogen atoms to the 3D structure of EST2-S35C, the AutoDock Tools (ADT) software package was used [32]. A box of about 90 Å^3^ was used to include the catalytic protein pockets. The 3D structures of paraoxon, parathion and diazinon were retrieved from the online database PubChem (https://pubchem.ncbi.nlm.nih.gov/ (accessed on 7 December 2021)) and used individually as the ligand of the EST2-S35C receptor. The protein was considered rigid during the docking procedure due to the thermophilic proteins’ rigidity at room temperature. The structures were analysed, and the images were produced using PyMOL [29] molecular graphics software.

## 3. Results and Discussion

### 3.1. EST2-S35C Purification and Labelling

The over-expression and biochemical characterisation of EST2-S35C were described in detail in Carullo et al. [23], confirming that the serine-to-cysteine residue mutation does not affect the catalytic site structure, and the structure–function relationship is unchanged. In addition, similarly to EST2, EST2-S35C remains sensitive to paraoxon inhibition [23]. Furthermore, using a single chromatographic step of molecular-sieve chromatography, it returns a more active and stable protein, because EST2-S35C is subjected to fewer noticeable electrostatic and chemical impairments after ion-exchange or affinity chromatography.

In order to improve the efficiency of the IAEDANS labelling in the EST2-S35C, we incubated the protein in the presence of increasing molar excesses of the fluorescent probe, from 10- to 200-fold, and evaluated the fluorescence intensity of the conjugated IAEDANS. We observed the best binding efficiency at a protein:probe ratio of 1:100 (Table S1), and this ratio was therefore used for all the other experiments.

In accordance with Carullo et al. [23], the activity of EST2-S35C conjugated with IAEDANS remained unchanged with respect to the free enzyme, and it continued to be fully inhibited by paraoxon in a 1:1 ratio (Figure S1).

### 3.2. Fluorescence Resonance Energy Transfer (FRET) Measurements

**Figure 1:**
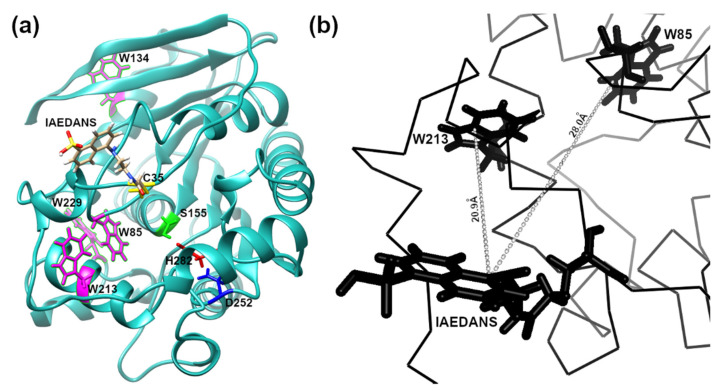
In silico representation of the 3D structure of the EST2-S35C: (**a**) ribbon representation showing in stick form: the catalytic triad Ser155 (green), Asp252 (blue) and His282 (red); the fluorescent probe IAEDANS; the tryptophan residues (magenta); (**b**) representation focusing on a part of the EST2-S35C backbone, highlighting the distance between the tryptophan residues 85 and 213.

**Figure 2:**
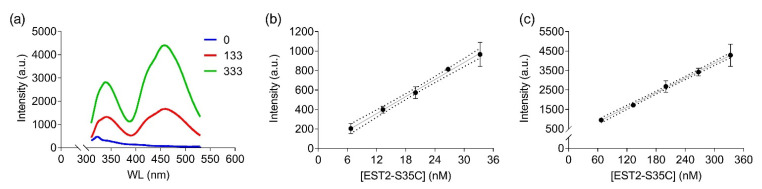
FRET measurements of labelled EST2-S35C after excitation at 290 nm: (**a**) illustrative FRET fluorescence spectra for 0, 133 and 333 nM of EST2-S35C; (**b**,**c**) fluorescence intensity (arbitrary units, mean ± SD, *n* = 3) at the maximum of emission (457–460 nm) for increasing EST2-S35C concentrations (low and high ranges, respectively).

In order to apply FRET to detecting low-concentration analytes, such as OP in solution, using the EST2-S35C mutant labelled with a fluorescent probe, we analysed the 3D structure of the protein in silico. The cysteine 35 is located in a loop near the entrance of the alcohol-binding pocket of EST2, fully exposed to binding with the IAEDANS (Figure 1a). The high accessibility on the protein surface of the cysteine 35 residue places the fluorescent probe outside the protein surface. In agreement with the Förster critical distance value, typically falling within a range of 20 to 60 Å, the fluorescent probe is close enough (between 20 and 30 Å) to the tryptophan residues 85 and 213 to enable resonance energy transfer to occur (Figure 1b). As expected, we observed an increase in the maximum fluorescence emission at 457–460 nm after tryptophan excitation at a wavelength of 290 nm (Figure 2a), confirming the energy transfer from these residues to the fluorescent probe. A significant linear increase in the fluorescence intensity of IAEDANS after energy transfer from tryptophan was observed for both low (Y = 29.12X + 8.916, R^2^ = 0.99, F_(1,3)_ = 899.9, *p* < 0.0001, Figure 2b) and high (Y = 12.57X + 98.1, R^2^ = 0.99, F_(1,3)_ = 2437, *p* < 0.0001, Figure 2c) protein amounts. Our results agree with previous results, where linear behaviour of the labelled EST2-S35C was obtained with different fluorescence-based methods [33].

### 3.3. pH Influence on FRET Measurements

**Figure 3:**
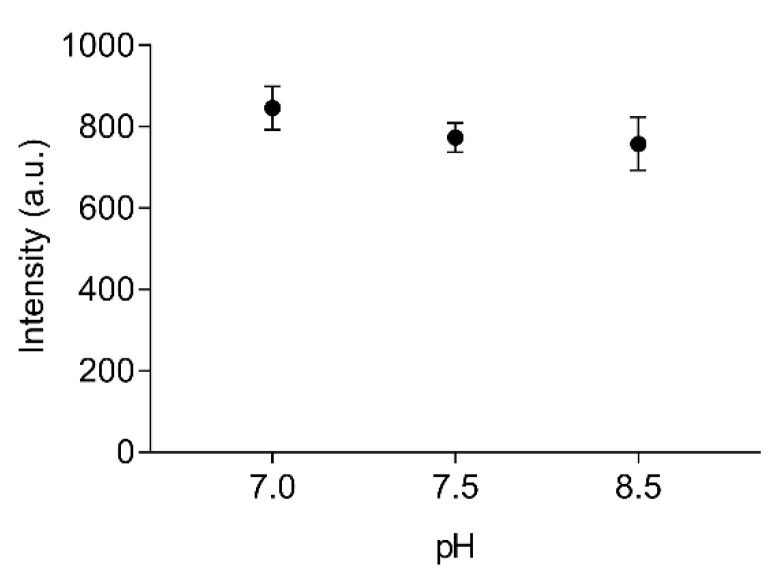
Fluorescence transfer intensity (arbitrary units, mean ± SD, *n* = 3) between the tryptophan residues and the IAEDANS of 67 nM of labelled EST2-S35C, using 25 mM NaH_2_PO_4_ at pH 7.0 and 25 mM Tris/HCl at pH 7.5 and 8.5 as media.

The stability of labelled EST2-S35C at different pH values was tested in the range from 7.0 to 8.5, which covers important liquid samples such as blood (pH 7.4) and drinking water (pH 7.0). We used two buffers with different properties. In particular, Tris/HCl is a widely used buffer suitable for the solubilisation of molecules with organic properties, while the inorganic NaH_2_PO_4_ is more appropriate for ion solubilisation. No significant differences (F_(2,6)_ = 2.36, *p* = 0.18, R^2^ = 0.44) were observed when applying FRET for measuring the fluorescence intensity of 67 nM labelled EST2-S35C (Figure 3). The pH is one of the major factors that can imply changes in enzyme conformations, impairing their activity. These results are not surprising, since EST2 is known to tolerate a certain range of pH fluctuations without compromising its activity [18]. However, due to the moderate to extremely acidic nature of fruit juices, any measurements of these samples must be performed in a buffered solution.

### 3.4. Effects of Organic Compounds on FRET Measurements

**Figure 4:**
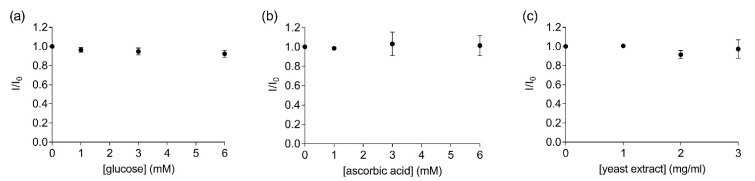
Ratios of the fluorescent intensity (mean ± SD, *n* = 3) of 67 nM labelled EST2-S35C at the maximum of emission (457–461) in the presence (I) and the absence (I_0_) of different concentrations of the tested substances: (**a**) glucose (mM); (**b**) ascorbic acid (mM); (**c**) yeast extract (mg/mL).

Another factor that must be considered when developing a biosensor for field applications is the complexity of the components present in the liquid sample. For instance, complex samples such as fruit juices can contain a variety of other molecules such as sugars, vitamins and other proteins. Therefore, measurements were performed to determine the possible interference of other non-target organic compounds in solution in the FRET measurements. No significant changes in the fluorescence ratios were observed when increasing concentrations of glucose (Y = −0.012X + 0.987, R^2^ = 0.89, F_(1,2)_ = 15.32, *p* = 0.06, Figure 4a), ascorbic acid (Y = 0.004X + 0.997, R^2^ = 0.33, F_(1,2)_ = 0.99, *p* = 0.42, Figure 4b) or yeast (Y = −0.017X + 0.999, R^2^ = 0.28, F_(1,2)_ = 0.78, *p* = 0.47, Figure 4c) were added to the labelled EST2-S35C solution (67 nM). The replicability of the FRET measurements and the stability of the bioreceptor in the solutions containing the different organic compounds were good, with RSD values ranging from 3.36 to 4.63% (Table S2). Previous experiments evaluated the EST2 activity in solutions containing serum from juice fruit, detecting some interference at concentrations as low as 5% of organic load [18]. Thus, our data suggest that directly measuring the FRET between the tryptophan residues and the extrinsic probe may help to overcome these issues. In agreement with our results, Wu et al. [34] did not detect any interference of glucose in FRET measurements using the activity of a cholinesterase as the bioreceptor.

### 3.5. FRET Quenching by Paraoxon Addition

**Figure 5:**
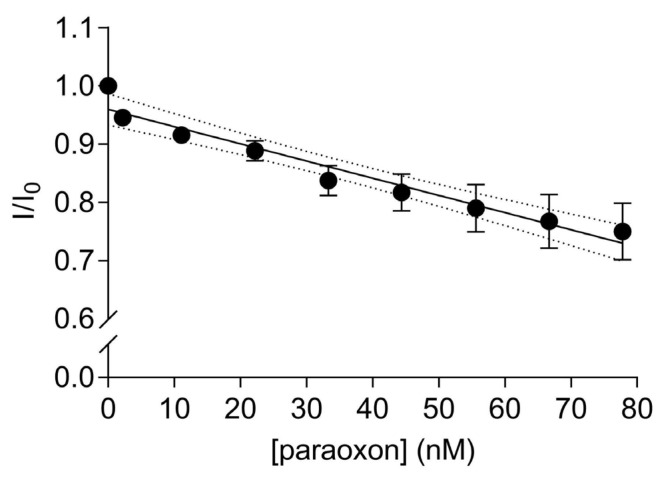
Ratios of the fluorescence intensity (mean ± SD, *n* = 3) of 67 nM IAEDANS-labelled EST2-S35C in the presence (I) and the absence (I_0_) of increasing concentrations (nM) of paraoxon at a maximum wavelength of emission of 458–460 nm. Dotted lines represent the 95% confidence interval for the linear regression.

A significant linear decrease in the fluorescence transfer was observed with increasing amounts of paraoxon addition to the solution containing 67 nM of the labelled EST2-S35C (Y = −0.002953X + 0.9597, R^2^ = 0.95, F_(1,7)_ = 129.6, *p* < 0.0001, Figure 5). The fluorescence is quenched due to the paraoxon binding in the catalytic site of EST2 and decreasing the FRET between the EST2 tryptophan donor and probe IAEDANS acceptor. As previously shown [21], the addition of paraoxon to the enzyme solution does not change the micro-environment of the tryptophan residues. Thus, we can exclude the interference of nonspecific interactions and associate the quenching of the fluorescence transfer with the steric bulk of the pesticide in the EST2-S35C catalytic site. The high precision and reproducibility of the paraoxon detection using FRET are demonstrated by the low values of RSD, ranging from 0.38 to 6.01% (Table S2). These values are similar to values obtained using classical techniques for pesticide residues detection, such as GC-MS [13]. A LOD of 0.57 nM and a LOQ of 2.0 nM were derived, though it should be taken into account that the dynamic linear range starts from 2.0 nM paraoxon. These values agree with our previous studies using fluorescence-based methods to evaluate EST2 sensitivity towards paraoxon [19] and are comparable with those of biosensors based on other methodologies. In fact, Zhang et al. proposed an AChE-based biosensor immobilised on a functionalised graphene oxide electrode, obtaining a LOD for paraoxon of 0.65 nM [35]. Similarly, Lang et al. obtained a LOD for paraoxon of 0.70 nM, using a sensitive amperometric AChE biosensor based on gold nanorods [36]. Arduini et al. evaluated the use of an electrochemical biosensor, using butyrylcholinesterase as the biosensor to detect paraoxon, and achieved a limit of detection of 22 nM and a linear range observed between approximately 70 and 360 nM [37]. The same study evaluated the recovery of paraoxon from a spiked biological matrix using the biosensor and high-performance liquid chromatography (HPLC), obtaining comparable recovery values of 60 and 63%, respectively. Combining nanomaterials with enzyme inhibition methods has also been an important topic of research. For instance, Zhai et al. [38] reviewed the use of Au nanomaterials decorated with enzymatic bioreceptors and found that limits of detection as low as 1 pmol/L were frequently reported. Nevertheless, the more complex technology involved in this type of biosensor makes it more expensive than the one proposed in this study.

### 3.6. EST2-S35C Specificity towards Paraoxon Detection by FRET Measurements

**Figure 6:**
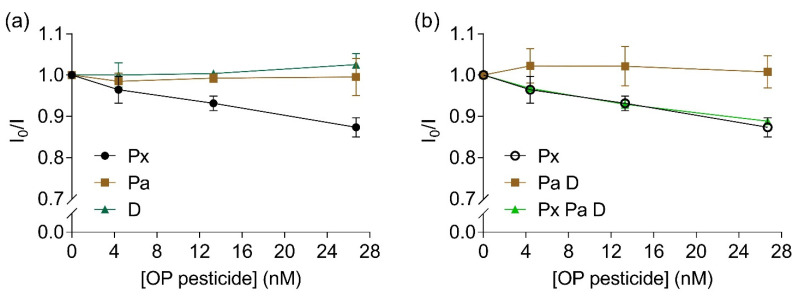
Ratios between the fluorescent intensity (mean ± SD, *n* = 3) of 67 nM IAEDANS-labelled EST2-S35C in the presence (I) and the absence (I_0_) of increased concentrations (nM) of: (**a**) individual OPs paraoxon (Px, maximum of emission 458–462 nm), parathion (Pa, maximum of emission 452–462 nm) and diazinon (D, maximum of emission 451–462 nm); (**b**) paraoxon (repeated for comparison purposes), mixture of 1:1 parathion:diazinon (maximum of emission 458.5–462.5 nm) and mixture of 1:1:1 paraoxon:parathion:diazinon (maximum of emission 459–462.5 nm).

To evaluate the selectivity of the bioreceptor towards paraoxon, the influence of other pesticides such as the thio-OPs parathion and diazinon were investigated. A linear inhibition of fluorescence intensity ratio was observed with increased concentrations of paraoxon (Y = −0.005X + 0.993, R^2^ = 0.99, F_(1,2)_ = 135.5, *p* = 0.007, Figure 6a). No significant alterations in fluorescence intensity ratios were observed for parathion (Y = 3.146e − 0.005X + 0.993, R^2^ = 0.003, F_(1,2)_ = 0.007, *p* = 0.94) or diazinon (Y = 0.001X + 0.997, R^2^ = 0.88, F_(1,2)_ = 14.33, *p* = 0.06, Figure 6a). When a mixture of parathion and diazinon was added to the bioreceptor, no alterations were measured regarding fluorescence intensity ratio (Y = 4.038e − 0.005X + 1.012, R^2^ = 0.002, F_(1,2)_ = 0.004, *p* = 0.95, Figure 6b, brown squares). When a mixture of all three studied pesticides were added to the solution with the labelled EST2-S35C, a significant linear inhibition was observed with increasing concentrations of pesticides (Y = −0.004X + 0.991, R^2^ = 0.97, F_(1,2)_ = 66.21, *p* = 0.01, Figure 6b, green triangles). Similarly to other cholinesterases, EST2 presents lower affinity towards thio-OPs, which explains the lack of FRET quenching observed at the tested levels.

Wu et al. [34] reported a FRET-based sensor to determine paraoxon using carbon quantum dots encompassing the inhibition of the butyrylcholinesterase activity, with a LOD of 0.2 nM. Similarly, Yan et al. [39] presented a FRET-based approach using a butyrylcholinesterase–acetylthiocholine–MnO_2_–carbon dots system for paraoxon detection ranging from 0.2 to 18 nM with a detection limit of 0.05 nM. CdTe-quantum-dot-based FRET sensors, using porphyrin bound to the surface of CdTe, reached a detection limit of 0.032 nM for paraoxon under optimal conditions [40], but with low specificity. FRET-based biosensors have also been used to detect other OPs, such as diazinon. Using aptamers conjugated with graphene oxide, different authors obtained a limit of detection for diazinon of 0.13 nM [41] and 0.08 nM [42]. Other FRET-based non-enzymatic sensors have been used for malathion detection. Exploiting the quenching in the FRET between a chemical fluorescent probe (energy donor) and β-cyclodextrin-coated silver nanoparticles (receptor), Wang et al. [43] obtained a limit of detection of 36 nM. Similarly, Chen et al. [44], using a fluorescent aptasensor and cationic-polymer-encapsulated gold nanoparticles, obtained a detection limit of 1.42 nM for malathion.

Compared to these FRET-based biosensors, our study has the advantage of presenting a simpler system that is less expensive and more specific, with a comparable detection limit in the nM range.

### 3.7. In Silico Analysis

The absence of competitive effects between the thio-OPs and the paraoxon was investigated using an in silico approach. In particular, we performed docking measurements to evaluate their placement in the protein and the extent of their influence on the energy transfer from tryptophan residues and the IAEDANS. Two binding pockets were experimentally identified in the EST2 structure [45]. The main one accommodates the acyl chains, while the secondary one recognises the alcohol groups (Figure S2). After minimisation, the docking analysis was performed on the EST2-S35C structure (with the cysteine mutation) obtained from the CHARMM-GUI online platform. The structures obtained from at least three different predictions for each OP ligand located the paraoxon in both the acyl- and alcohol-binding sites of the mutated protein, with a better affinity for the acyl site (Table 1). In contrast, parathion and diazinon were exclusively located in the alcohol-binding site (except for two wrong locations for diazinon, with a very low affinity of ≤4.6 kcal/mol in the opposite part of the protein in just one prediction), with the best affinity values in the range from −6.2 to −6.1 and −6.2 to −5.2 kcal/mol, respectively (Table 1).

These results are in agreement with a similar docking analysis carried out on the EST2 3D structure, which highlighted the presence of paraoxon in both catalytic pockets and diazinon in the alcohol-binding pocket only [21]. However, parathion was previously predicted to bind in both catalytic pockets. Thus, we can hypothesise that the mutation and the protein minimisation result in a different prediction.

However, these predictions are in complete accordance with the FRET measurements, supporting the different results observed for paraoxon (fluorescence quenching). In fact, only paraoxon was predicted to bind in the acyl pocket, affecting the energy transfer from Trp 85 to the IAEDANS probe. In contrast, the two thio-OPs (parathion and diazinon), although showing similar affinity for binding to EST2-S35C, did not affect the FRET because they were located only in the alcohol-binding site.

## 4. Conclusions

In this work, we demonstrated the use of FRET measurements to improve the efficiency of paraoxon detection. Our findings suggest that the binding of fluorescent probes, such as IAEDANS, near the alcohol-binding site, does not affect the enzyme’s binding or function in the acyl-binding site. More importantly, we achieved two interesting goals: (i) to reduce the interference in the fluorescence measurements due to the presence of other organic molecules in complex solutions; (ii) to increase the protein specificity, as the use of FRET measurements permitted observation of changes affecting only the acyl-binding pocket, strongly reducing possible interferences from nonspecific interactions at the alcohol-binding site.

In conclusion, this study paves the way to applying this bioreceptor for FRET measurements of real samples such as food and/or biological fluids, eventually combined with solid-state fluorescence measurements [33].

## Figures and Tables

**Table 1:** OPs best affinity values for each of the predicted binding pockets in EST2-S35C 3D structure.

Compound Name	Affinity (kcal/mol)	Pocket Binding
Paraoxon	−6.5 to −6.1	acyl
	−6.1 to −5.6	alcohol
Parathion	n.d.	acyl
	−6.2 to −6.1	alcohol
Diazinon	n.d.	acyl
	−6.2 to −5.2	alcohol

## Data Availability

Data is contained within the article or Supplementary Material. Further details may be made available under request to the authors.

## Supplementary Materials

The following are available online at https://www.mdpi.com/article/10.3390/s22020561/s1. Figure S1: Plot of residual activity of EST2-S35C conjugated with IAEDANS against increasing concentrations of paraoxon; Figure S2: Mesh representation of the cavities inside of the EST2-S35C which shape the catalytic site; Table S1: Fluorescence intensity of the IAEDANS probe after coupling reaction at different EST2-S35C:IAEDANS ratios.; Table S2: Relative standard deviation (RSD) of the performed FRET measurements.
