# Supplementary material for: A FRET Approach to Detect Paraoxon among Organophosphate Pesticides Using a Fluorescent Biosensor
Source: Sensors (Basel). 2022 Jan 12;22(2):561. doi: 10.3390/s22020561 (PMC8778994; doi:10.3390/s22020561)
Supplement: Supplementary file 1 [file sensors-22-00561-s001.zip › sensors-1523694-supplementary.pdf]

sensors

MDPI

Supplementary Materials

A FRET Approach to Detect Paraoxon among Organophosphate Pesticides Using a Fluorescent Biosensor

Andreia C. M. Rodrigues [1,*,†], Maria Vittoria Barbieri [1,†], Marco Chino [2], Giuseppe Manco [1,‡] and Ferdinando Febbraio [1,*,‡]

[1] Institute of Biochemistry and Cell Biology, CNR, Via P. Castellino, 111, 80131 Naples, Italy; mariavittoria.barbieri@ibbc.cnr.it; giuseppe.manco@cnr.it;
[2] Department of Chemical Sciences, University of Naples "Federico II", 80126 Naples, Italy; marco.chino@unina.it
* Correspondence: rodrigues.a@ua.pt (A.C.M.R.); ferdinando.febbraio@cnr.it (F.F.)
† These authors contributed equally to this work.
‡ These authors contributed equally to this work.

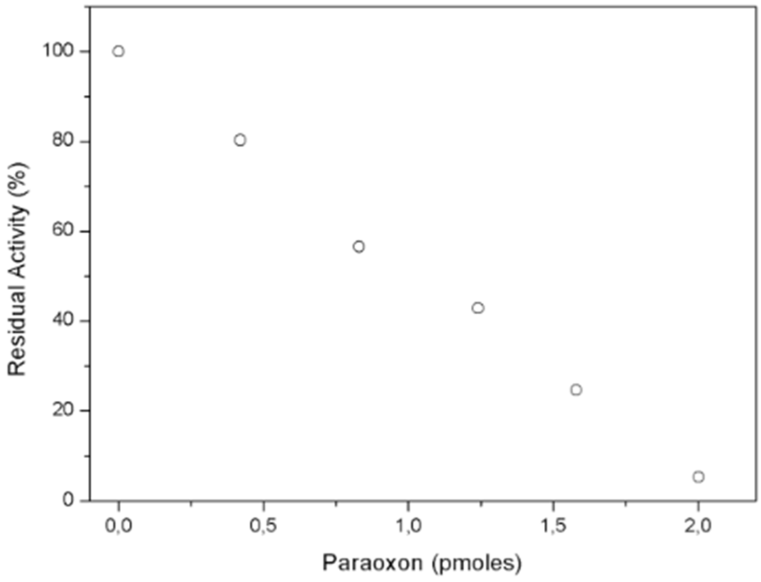

Supplementary Figure S1. Plot of residual activity of EST2-S35C conjugate with IAEDANS against increasing concentrations of paraoxon in the range from 0 to 2 pmol.

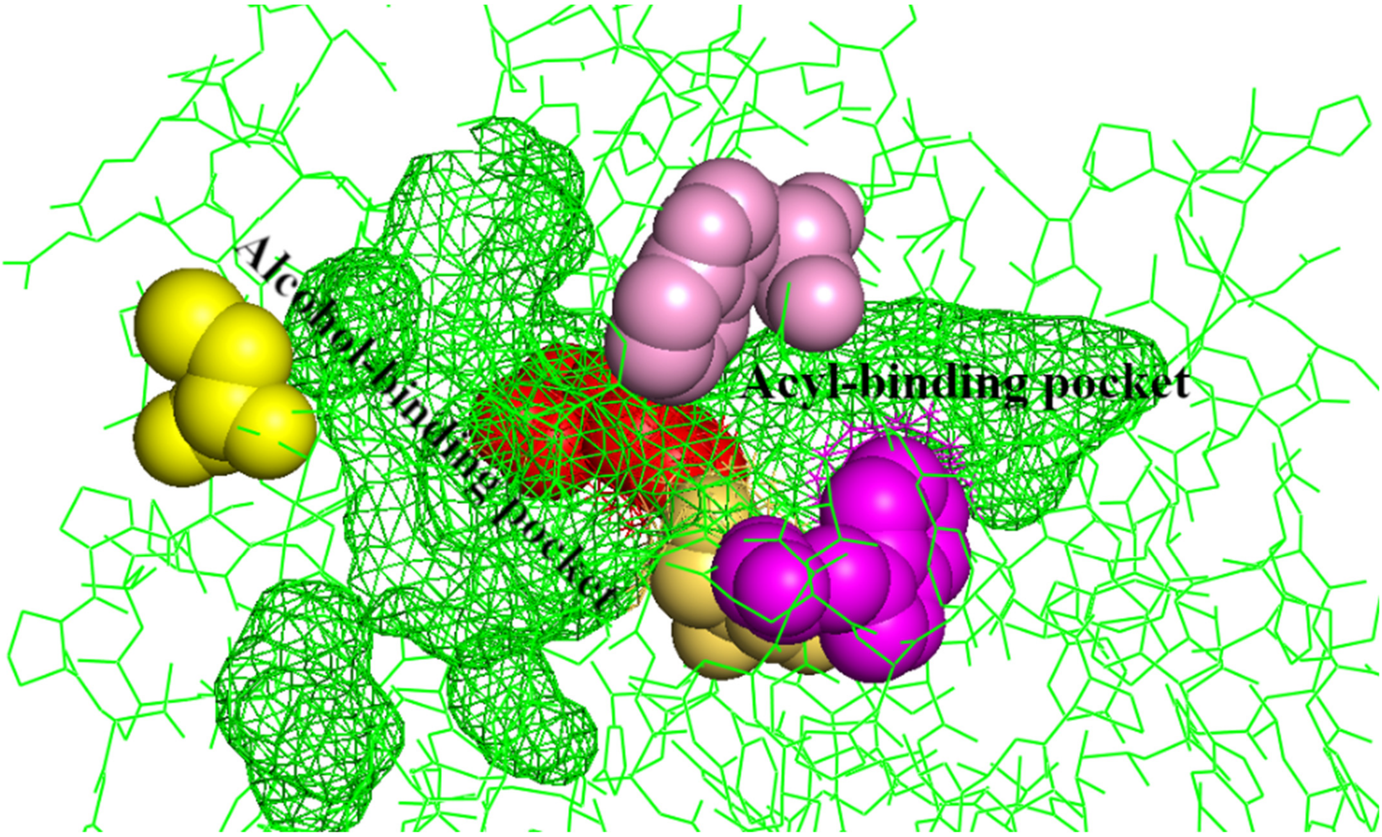

Supplementary Figure S2. Mesh representation of the cavities inside of the EST2-S35C which shape the catalytic site. The acyl- and alcohol-binding pockets and the residues of Cys35 (yellow), Ser155 (orange-yellow), His282 (red), Trp85 (magenta) and Trp 213 (pink) are indicated.

Supplementary Table S1. Fluorescence intensity of the IAEDANS probe after coupling reaction at different EST2-S35C:IAEDANS ratios.

EST2-S35C:IAEDANS Ratio	Wavelength (nm)	Intensity (Arbitrary Units)
1:10	455	195.84
1:50	457	546.09
1:100	457.5	1726.00
1:200	457.5	1084.54

Supplementary Table S2. Relative standard deviation (RSD) of the performed FRET measurements.

Data Reference	Treatment			RSD (%)
	EST2-S35C (pmol)	Compound	(pmol)	
Figure 2b	3			25.44
	6			8.75
	9			10.44
	12			2.72
	15			12.74
Figure 2c	30			1.20
	66			3.26
	90			11.16
	120			5.65
	150			13.30
Figure 3	30	pH 7.0		6.33
		pH 7.5		4.63
		pH 8.5		8.65
Figure 4a	30	Glucose		3.36
Figure 4b	30	Ascorbic acid		4.63
Figure 4c	30	Yeast		4.29
Figure 5	30	Paraoxon	1	0.38
			5	0.71
			10	1.94
			15	3.05
			20	3.89
			25	5.15
			30	6.01
			35	6.50
Figure 6a	30	Paraoxon	2	3.36
			6	1.89
			12	2.66
		Parathion	2	1.99
			6	1.03
			12	4.51
		Diazinon	2	2.94
			6	0.28
Figure 6b	30	Parathion + Diazinon	2	2.58
			6	4.09
			12	4.68
		Paraoxon + Parathion + Diazinon	2	3.91
			6	0.83
			12	1.04
